# Differences in the Structure and Function of the Gut Microbiota of the Earthworm (*Amynthas aspergillum*) Under Different Habitat Conditions

**DOI:** 10.3390/life16101648

**Published:** 2026-09-30

**Authors:** Xuesong Li, Bo Lin, Lixin Peng, Yangmei Qin, Rongdian Ban

**Affiliations:** 1 College of Animal Science and Technology, Guangxi University, Nanning 530004, China; 2318391026@st.gxu.edu.cn (X.L.); dzq369@gmail.com (R.B.); 2 State Key Laboratory of Non-Food Biomass Energy Technology, Institute of Biological Science and Technology, Guangxi Academy of Sciences, Nanning 530007, China; penglixin@gxas.cn (L.P.); qinym@gxas.cn (Y.Q.)

**Keywords:** *Amynthas aspergillum*, gut microbiota, habitat heterogeneity, community assembly, functional prediction, ecological breeding

## Abstract

This study aims to explore the shaping mechanism of habitat heterogeneity on its gut microecology and to clarify the structural differences and functional succession patterns of gut microbiota of an earthworm *Amynthas aspergillum* under artificial breeding versus natural habitats. A total of 175 *A. aspergillum* specimens and their corresponding soil samples were collected from seven typical habitats in Guangxi: fertile farmland, barren farmland, plantation forest, primary forest, artificial grassland, wasteland slope, and a breeding farm. 16S rRNA high-throughput sequencing was employed to analyze the gut microbiota structure. PICRUSt2 was used to predict metabolic functions, and the correlation between soil physicochemical factors and community characteristics was analyzed. There were only 25 core shared OTUs found across the seven habitats, indicating a strong habitat specificity of the gut microbiota in *A. aspergillum*. The fertile farmland group exhibited the highest species richness (Chao1) and diversity (Shannon) among all groups (*p* < 0.05). Although rich in organic matter, the breeding farm group showed the highest Simpson dominance index. *Actinomycetota*, *Pseudomonadota*, Chloroflexota, and *Bacillota* were the dominant shared phyla in all groups. LEfSe analysis revealed that the wasteland slope habitat specifically enriched the stress-tolerant genus *Mycobacterium*, while the breeding farm was dominated by the anaerobic fermentation-capable genus *Clostridium*. Functional prediction indicated that the primary forest group had the most complex microbial metabolic network, while the breeding farm group exhibited a “functional simplification” trend focused on basic carbohydrate metabolism, which was significantly correlated with the soil C/N ratio. Soil physicochemical factors, particularly the intensity of anthropogenic disturbance and the composition of organic matter, are the key forces driving the assembly of *A. aspergillum* gut microbiota through environmental filtering. Artificial monoculture breeding leads to the functional degradation of the gut microecology.

## 1. Introduction

The gut microecosystem of soil animals serves as a critical functional determinant for host environmental adaptation, nutrient acquisition, and pathogen defense [1,2,3,4]. *Amynthas aspergillum*, belonging to the family Megascolecidae and commonly known as “Guangdilong,” is one of the most widely distributed and abundant large soil animals in South China, as well as a significant traditional Chinese medicine listed in the National Pharmacopoeia [5,6,7]. In recent years, wild resources of *A. aspergillum* have declined sharply due to habitat fragmentation and predatory harvesting, prompting the emergence of the artificial breeding industry [8,9,10]. However, current breeding models largely rely on high density and single substrates (e.g., cattle manure, agricultural waste). This often leads to issues such as germplasm degeneration, compromised disease resistance, and unstable yields. Accumulating evidence indicates that alterations in gut microbial compositions driven by external environments may partially account for these microecological complications [11]; however, the precise regulatory mechanisms shaping these habitat-dependent successions remain poorly understood [12].

The assembly of gut microbial communities in soil animals is synergistically regulated by the dual forces of “host genetic background” and “environmental filtering” [13,14,15]. For earthworms, gut microbiota are primarily derived from ingested surrounding soil; however, the ingested microbiota undergo intense directional selection after entering the earthworm’s gut-specific microenvironment (characterized by neutral pH, anaerobic conditions, and high mucus secretion). This process ultimately results in a unique community structure that is distinct from the external environment [16,17,18,19]. These gut-colonizing symbiotic microorganisms are not only key executors for nutrient acquisition and macromolecule degradation but also core functional components for resisting exogenous pathogens and maintaining host health and environmental adaptability [20,21,22]. There were many studies showing that there are core microbial communities existing in the guts of different animals; therefore, core gut microbial communities also exist in earthworm and persist stably and dominate under various environmental conditions and host states [23].

However, existing studies are mostly confined to static descriptions of the earthworm’s gut microbe composition in single environments or focus on unidirectional stress effects from specific pollutants (e.g., heavy metals, antibiotics). There is a lack of systematic analysis of effects of the “soil-earthworm-microbe” interaction on the earthworm’s gut microbial community, especially the core microbiota, across a broad gradient of habitats—ranging from natural ecosystems to intensive artificial ecosystems. Specifically, how soil physicochemical properties (e.g., pH, organic matter, C/N ratio) in different habitats reshape gut microbiota through cascade effects, and whether artificial monoculture environments lead to functional alienation or simplification of the gut microecology, are urgent issues that directly impact the sustainable development of the ecological breeding industry [24].

This study investigates gut relative and absulate bacterial composition of earthworm of seven typical habitats in subtropical area, covering a gradient from natural (primary forest) and semi-natural (plantation forest, wasteland slope) to artificial (farmland, breeding farm) ecosystems by using 16S rRNA gene high-throughput sequencing method combined with bioinformatics analysis. This study aims to: (1) reveal the driving characteristics of habitat heterogeneity on the diversity and community structure of *A. aspergillum* gut microbiota; (2) identify core indicator taxa (biomarkers) across different habitats; (3) elucidate the potential mechanisms of environment-induced functional succession of the microbiota. The findings will provide a theoretical basis for the conservation of “Guangdilong” germplasm resources, habitat restoration, and the optimization of ecological breeding techniques.

## 2. Materials and Methods

### 2.1. Study Area and Sampling Sites

**Figure 1:**
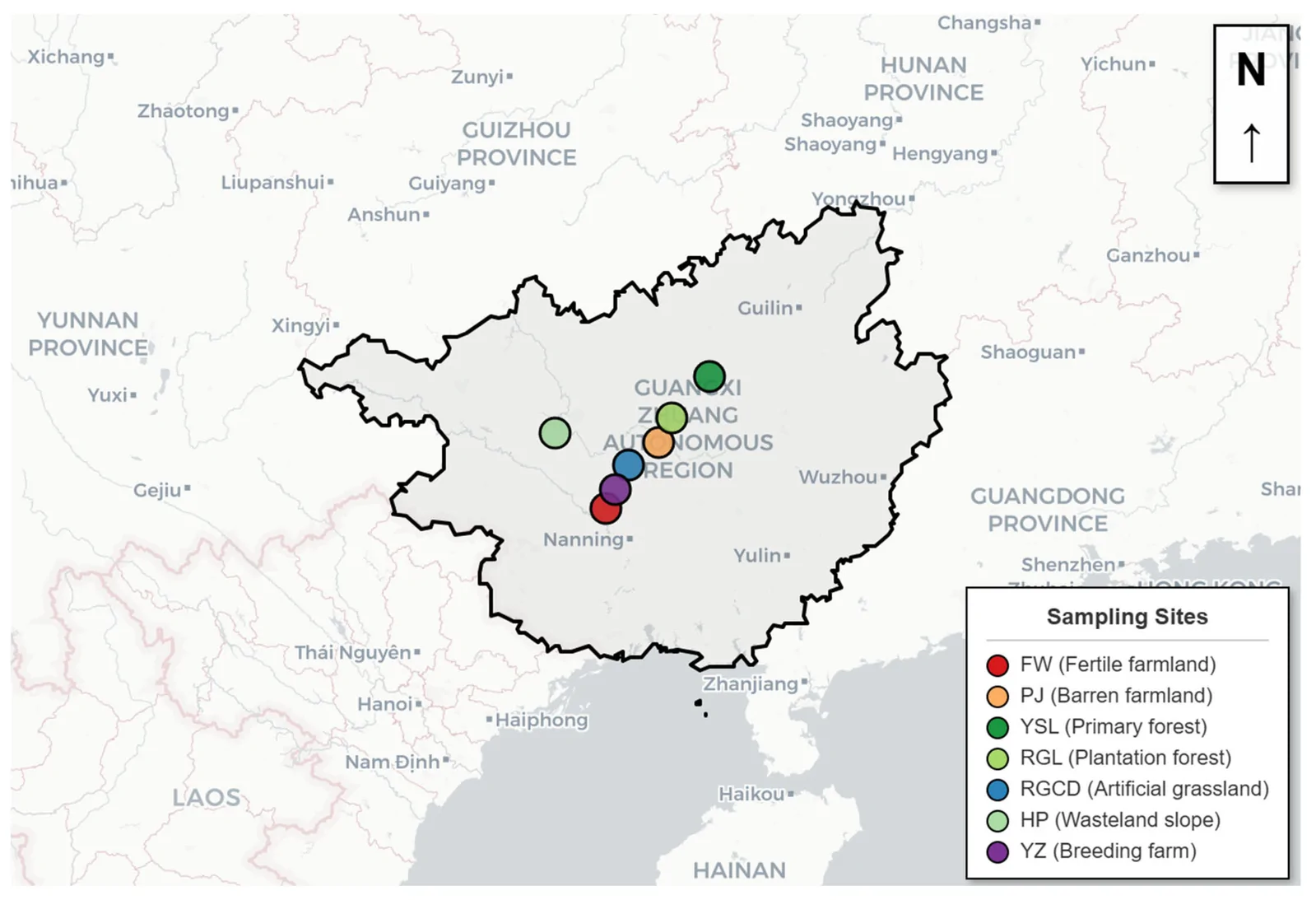
Geographical distribution of the sampling sites for the earthworm *Amynthas aspergillum* in Guangxi, China. Colored dots represent different habitat types: FW, fertile farmland; HP, wasteland slope; PJ, barren farmland; RGCD, artificial grassland; RGL, plantation forest; YSL, primary forest; YZ, breeding farm.

The study area is located in the Guangxi Zhuang Autonomous Region (21.0° N–26.0° N, 104.0° E–112.0° E) (Figure 1), which is characterized by a subtropical monsoon climate [25,26]. To systematically investigate the characteristics of earthworm gut microbiota across different habitats, seven typical habitats were selected based on vegetation type, anthropogenic disturbance intensity, and soil fertility: fertile farmland (FW), barren farmland (PJ), primary forest (YSL), plantation forest (RGL), artificial grassland (RGCD), wasteland slope (HP), and a breeding farm (YZ). Notably, the substrate at the breeding farm site consisted of a mixture of fermented cattle manure and straw.

The gradient of anthropogenic disturbance intensity was rigorously classified into three developmental tiers: natural control undisturbed (YSL), semi-natural low-intensity disturbed (RGL, HP), and high-intensity artificial continuous managed intervention (FW, PJ, RGCD, YZ).

### 2.2. Earthworm and Soil Sample Collection

**Figure 2:**
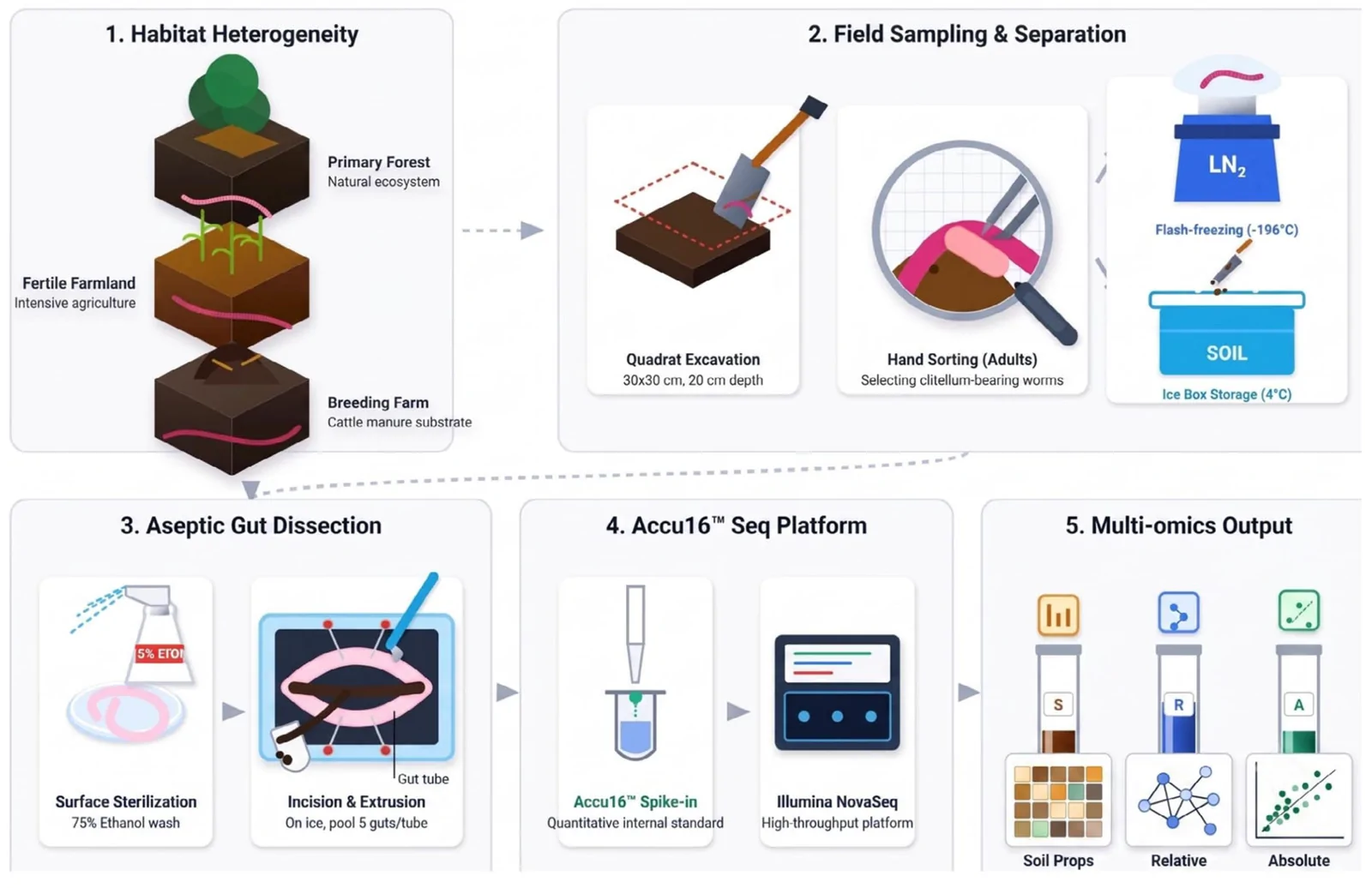
Comprehensive workflow diagram of habitat-specific sampling, aseptic extraction, and absolute quantification for the gut microecology of the earthworm *Amynthas aspergillum*.

In each habitat, a multi-point sampling strategy with five random quadrats (30 cm × 30 cm) was employed to collect earthworms [27]. The soil was excavated vertically to a depth of 20–30 cm to collect healthy, sexually mature adult *Amynthas aspergillum*. Active earthworms were captured from these multi-point quadrats, and 25 healthy individuals were pooled together as a representative composite sample for each habitat, totaling 175 specimens. Field sampling was conducted in mid-October 2025 (autumn season). At the breeding farm (YZ) site, the mixed substrate of fermented cattle manure and straw had been fully matured and stabilized for approximately 45 days prior to sampling to ensure a stable colonizing microenvironment for the earthworms. The earthworms used for sampling had an average body weight of 9.50 ± 0.85 g and a body length of 14.00 cm to 18.50 cm. The sampled earthworms were directly frozen in liquid nitrogen and taken back to the laboratory. As for the soil samples, they were collected from a depth of 0–20 cm within a radius of 10–15 cm around the earthworm discovery points. For each habitat, five soil samples were obtained following the methods of sample collection, processing, and analysis described by Bolyen et al. [28]. All samples were immediately placed in ice boxes and returned to the laboratory for processing as soon as possible. The sampling procedure was shown in Figure 2.

### 2.3. Determination of Soil Physicochemical Properties

Soil samples were air-dried in the shade and passed through a 2 mm sieve. To ensure maximum scientific rigor and comply with international standards, the physicochemical properties were determined based on standard laboratory analytical protocols. Briefly, soil pH was measured using a digital pH meter with a 1:2.5 soil-to-water suspension ratio; total nitrogen (TN) was determined via the classic Kjeldahl digestion method; total phosphorus (TP) was analyzed using the molybdenum blue colorimetric method; and organic matter (OM) was measured by the potassium dichromate oxidation-outer heating method. The results are expressed as the mean ± standard error (SE) of replicates within each habitat group, and one-way analysis of variance (ANOVA) was performed for statistical analysis.

Note that while transient soil moisture and microclimate fluctuations were not individually presented, all field macro-environmental operations were synchronized within the same stable meteorological window to ensure background homogeneity.

### 2.4. Earthworm Sample Processing and DNA Extraction

Gut Content Collection: The earthworm body surfaces were cleaned with sterile water and disinfected with 75% ethanol. After being anesthetized on ice, the earthworms were aseptically dissected to extract the intestines, and the gut contents were collected by extrusion. Gut contents from five earthworms were pooled to form one biological replicate. Five replicates were obtained for each habitat, resulting in a total of 35 samples. The samples were flash-frozen in liquid nitrogen and stored at −80 °C.

DNA Extraction: Total DNA was extracted from the gut content samples using the DNeasy PowerSoil Pro Kit (Qiagen, Hilden, Germany) and purified with VAHTS DNA Clean Beads (Vazyme Biotech Co., Ltd., Nanjing, China). DNA quality and concentration were assessed via agarose gel electrophoresis and a Qubit 4.0 Fluorometer (Thermo Fisher Scientific, Waltham, MA, USA). For each biological replicate, approximately 0.25 g of pooled gut content was used for total genomic DNA extraction.

### 2.5. Relative and Absolute Quantification of 16S rRNA Genes Based on Spike-In Internal Standards

This study employed Accu16™ technology to perform dual-dimensional sequencing analysis (relative and absolute). Using qualified gut DNA as a template, a known copy number of exogenous synthetic DNA (Spike-in control) was quantitatively added as an internal reference prior to PCR amplification. Subsequently, the V3–V4 region of the 16S rRNA gene was amplified using primers 338F/806R to construct libraries, which were then subjected to high-throughput sequencing on the Illumina NovaSeq 6000 platform (Illumina Inc., San Diego, CA, USA) using a paired-end (2 × 250 bp) sequencing strategy with the NovaSeq Reagent Kit (Illumina Inc., San Diego, CA, USA). During data processing, in addition to calculating the relative abundance composition of each Operational Taxonomic Unit (OTU) using standard methods, the “sequencing reads-to-known copy number” linear standard curve of the Spike-in internal standard was utilized. This allowed for the conversion of relative sequencing data for each OTU into absolute abundance (copies/g) per gram of gut content, thereby achieving synchronized and precise quantification of both community structure proportions and the true species load.

### 2.6. Bioinformatics Analysis

Raw sequencing data were first preprocessed to remove primers and adapters using Cutadapt (v1.18), followed by sequence splicing and quality control with FLASH (v1.2.11). Operational Taxonomic Units (OTUs) were clustered at a 97% similarity threshold using the UPARSE algorithm (USEARCH v10.0), and taxonomic annotation was performed against the Silva database (v138) [29]. To evaluate sequencing depth coverage for microbial diversity across all samples, rarefaction curves were generated using the alpha-rarefaction command in QIIME 2 (v2022.2).Venn diagrams illustrating the number of unique and shared OTUs among the seven habitat groups were constructed using the VennDiagram package in R, based on OTU presence/absence data from all samples within each group.

Alpha diversity indices, including Observed OTUs, Chao1 (richness), ACE, Shannon (diversity), Simpson (dominance), and Pielou_e (evenness), were calculated for each sample based on absolute quantification-corrected data, and results were visualized via boxplots. Inter-group differences were compared using the Kruskal–Wallis test (*p* < 0.05). For beta diversity analysis, the Bray–Curtis dissimilarity matrix was calculated using absolute abundance data to evaluate variation in community structure across samples. Non-metric Multidimensional Scaling (NMDS) was applied for ordination, and the Emperor tool (v2020.8) was used for visualization to demonstrate the separation of samples from different habitats in terms of community structure.

For community structure composition analysis, OTU sequence counts for each sample were normalized by Total Sum Scaling (TSS) to calculate the relative abundance of each OTU as a percentage of the total sequence count of the sample, which was used to generate stacked bar charts at the phylum and genus levels to display relative community composition. Absolute abundance calculation followed the internal standard method described in Section 2.5: sequencing reads of all OTUs in each sample were converted into absolute copy numbers (copies/g) using the Spike-in standard curve. At the phylum and genus levels, mean absolute copy numbers of samples within each habitat group were calculated as Mean ± SEM, and One-way ANOVA or Kruskal–Wallis tests were performed for inter-group comparisons. Differential indicator species were screened via LEfSe (Linear Discriminant Analysis Effect Size) analysis: the non-parametric Kruskal–Wallis test was first used to identify species with significant differences among groups, then Linear Discriminant Analysis (LDA) was applied to evaluate the effect size of each differential species. Species with an LDA score threshold > 3.5 and *p* < 0.05 were considered statistically significant biomarkers [30,31].

Functional prediction of the gut microbiota was conducted using PICRUSt2 software based on denoised representative sequences of OTUs. The EPA-ng and GAPPA algorithms were used to precisely place OTU sequences into a reference phylogenetic tree, and the Hidden State Prediction (HSP) method was applied to infer gene family copy numbers for each OTU. Weighted calculations were then performed to generate a predicted abundance table of KEGG Orthologs (KO) for each sample. KO abundances were mapped to the functional hierarchy (Level 1–3) of the KEGG database. To reveal habitat-specific differences, the Kruskal–Wallis H test was used to screen for Level 3 functional pathways with significant abundance differences (*p* < 0.05) among different habitat groups. Finally, the R package ggalluvial was used to draw Sankey diagrams illustrating functional flows, and the pheatmap package was used to perform Z-score normalization on the significantly different Level 3 pathways [32,33].

### 2.7. Statistic Analysis

All data of the soil and gut microbiota indicators were initially compiled in Excel and statistically analyzed using SPSS Statistics software (version 27.0, IBM Corp., Armonk, NY, USA). Mean values of corresponding indicators for replicate samples within each treatment group were first calculated, then One-way ANOVA was used to compare differences among different treatment groups. If ANOVA results showed significant differences (*p* < 0.05), Duncan’s multiple range test was further applied for pairwise comparisons between groups.

## 3. Results

### 3.1. Analysis of Differences in Soil Physicochemical Properties Across Seven Habitats

The soil pH, organic matter, total nitrogen, total phosphorus, total potassium, carbon-to-nitrogen (C/N) ratio, and electrical conductivity (EC) were determined for seven habitats: fertile farmland (FW), wasteland slope (HP), barren farmland (PJ), artificial grassland (RGCD), plantation forest (RGL), primary forest (YSL), and breeding farm (YZ) (Table 1). One-way ANOVA indicated highly significant differences in all indices among the groups (*p* < 0.001). Fertile farmland (FW) exhibited significantly higher contents of organic matter (29.12 g/kg), total nitrogen (2.10 g/kg), total phosphorus (0.98 g/kg), and total potassium (18.30 g/kg) compared to all other groups. In contrast, the wasteland slope (HP) recorded the lowest levels for all nutrient indices, with an organic matter content of 7.02 g/kg. The substrate at the breeding farm (YZ) displayed unique physicochemical characteristics, featuring the highest total phosphorus content (1.00 g/kg) and the lowest C/N ratio (9.38). The soil in the primary forest (YSL) was acidic (pH 5.34), whereas the soil in the wasteland slope (HP) was alkaline (pH 8.02). The highest C/N ratios were observed in the wasteland slope (HP) and primary forest (YSL), at 17.70 and 16.90, respectively. Furthermore, the highest electrical conductivity values were recorded in the wasteland slope (HP) and breeding farm (YZ), at 407.00 μS/cm and 320.00 μS/cm, respectively.

### 3.2. Evaluation of Sequencing Data and OTU Distribution Characteristics of Amynthas aspergillum Gut Microbiota Under Seven Habitat Conditions

**Figure 3:**
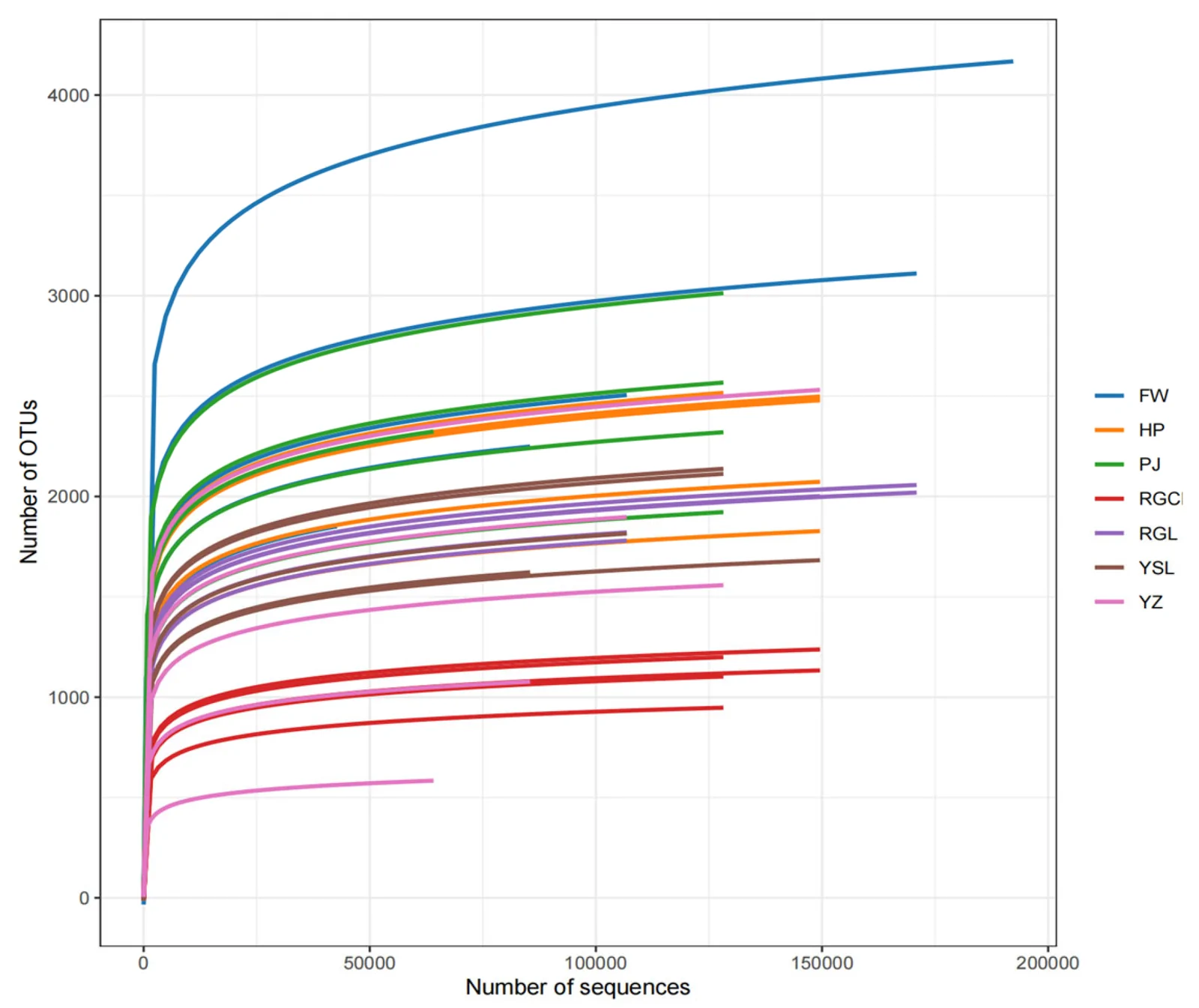
Rarefaction curves of earthworm gut samples from different habitats. FW, fertile farmland; HP, wasteland slope; PJ, barren farmland; RGCD, artificial grassland; RGL, plantation forest; YSL, primary forest; YZ, breeding farm.

**Figure 4:**
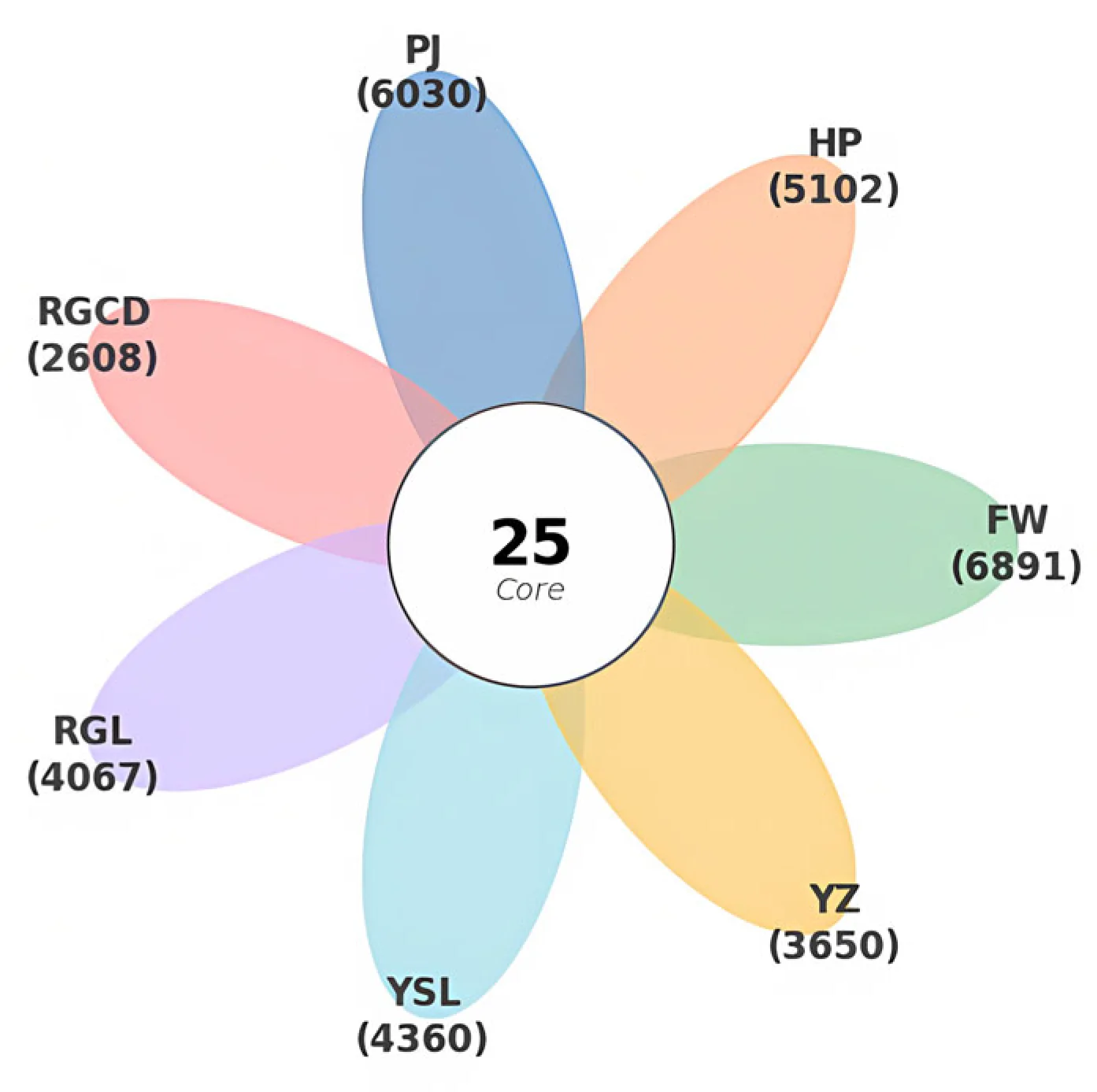
Venn diagram of shared and unique OTUs in earthworm gut microbiota across different habitats. FW, fertile farmland; HP, wasteland slope; PJ, barren farmland; RGCD, artificial grassland; RGL, plantation forest; YSL, primary forest; YZ, breeding farm.

In this study, clustering at a 97% similarity threshold yielded a total of 32,733 OTUs across all samples. Rarefaction curves indicated that the sequencing depth sufficiently covered the microbial diversity of each sample (Figure 3). Venn diagram analysis revealed that only 25 core OTUs (accounting for 0.08%) were shared among the seven habitat groups, whereas the number of unique OTUs varied significantly across groups. Specifically, the fertile farmland group (FW) possessed the highest number of unique OTUs (6891), while the RGCD group had the lowest (2608) (Figure 4). These findings suggest that habitat heterogeneity strongly shapes the unique taxa within the gut microbial community of *A. aspergillum*.

### 3.3. Alpha and Beta Diversity Analysis of Amynthas aspergillum Gut Microbiota Under Seven Habitat Conditions

**Figure 5:**
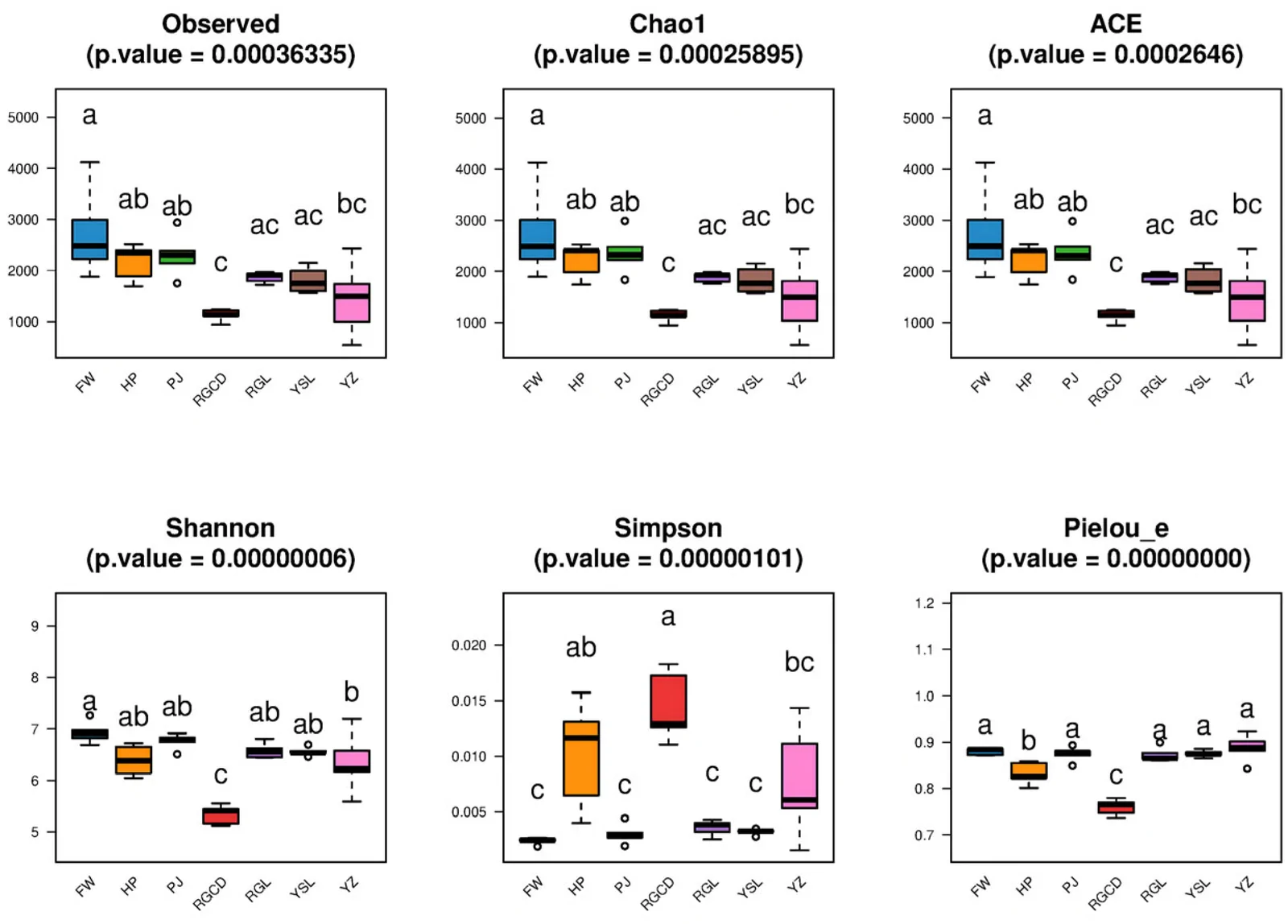
Alpha diversity indices of Amyntas aspergillum gut microbiota across different groups. FW, fertile farmland; HP, wasteland slope; PJ, barren farmland; RGCD, artificial grassland; RGL, plantation forest; YSL, primary forest; YZ, breeding farm. Different letters above the boxes indicate significant differences between groups (*p* < 0.05).

Absolute quantitative sequencing (Accu16S™) revealed that habitat heterogeneity profoundly drives gut microbial alpha diversity in *A. aspergillum*. Natural and semi-natural habitats—particularly fertile farmland (FW), wasteland slopes (HP), and barren farmland (PJ)—supported significantly higher species richness and diversity than artificial environments. Specifically, the FW microbiome exhibited peak richness (~2800 OTUs). In stark contrast, the intensively managed artificial grassland (RGCD) harbored the lowest richness (~1100 OTUs), alongside maximum community dominance (with a Gini-Simpson index value of ~0.015, representing peak dominance density) and minimum evenness (~0.75). While microbial evenness remained robust (0.88–0.90) across most other habitats (Figure 5), these results collectively demonstrate that highly simplified, artificial ecosystems severely deplete gut microbial diversity, forcing communities toward homogenization and monodominance.

**Figure 6:**
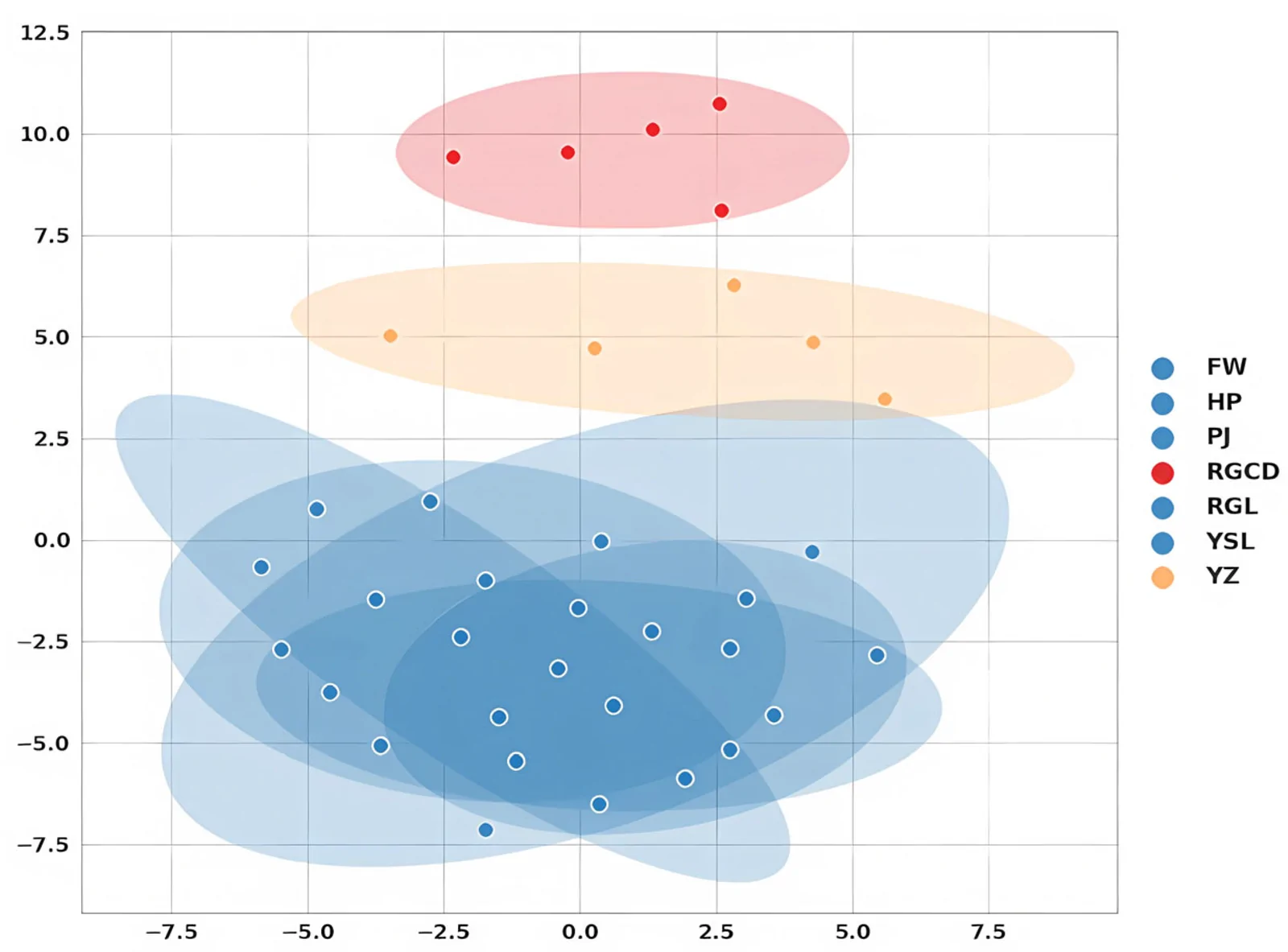
Non-metric Multidimensional Scaling (NMDS) plot of β-diversity for *Amyntas aspergillum* gut microbiota across different groups. FW, fertile farmland; HP, wasteland slope; PJ, barren farmland; RGCD, artificial grassland; RGL, plantation forest; YSL, primary forest; YZ, breeding farm.

NMDS ordination (Bray–Curtis, stress = 0.18) demonstrated that habitat type is a primary driver of compositional shifts in the *A. aspergillum* gut microbiota. The RGCD and YZ groups formed discrete, non-overlapping clusters, underscoring the profound impact of intensive anthropogenic management on microbial assembly (Figure 6). While the HP group occupied a distinct marginal position, the FW, PJ, YSL and RGL groups exhibited substantial spatial overlap (Figure 6). This compositional convergence suggests that natural and semi-natural environments maintain relatively consistent microbial structures, whereas highly artificialized habitats induce unique, divergent microbial signatures.

### 3.4. Analysis of Relative/Absolute Abundance of Gut Microbiota at the Phylum Level

**Figure 7:**
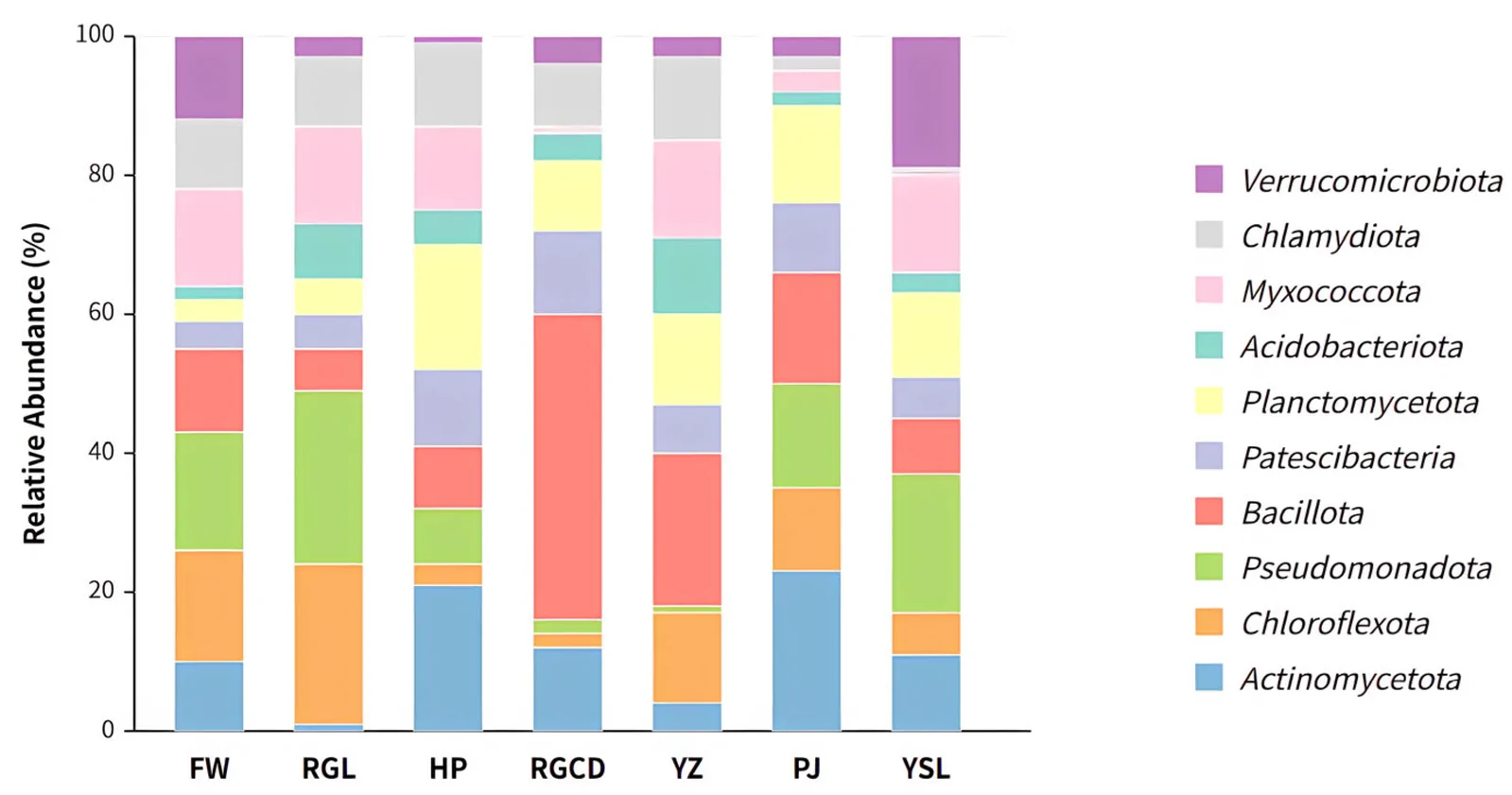
Relative abundance of major bacterial phyla in the gut microbiota of *Amyntas aspergillum* across different habitats. The stacked bar chart displays the top 10 most abundant phyla. FW, fertile farmland; HP, wasteland slope; PJ, barren farmland; RGCD, artificial grassland; RGL, plantation forest; YSL, primary forest; YZ, breeding farm.

At the phylum level, the gut microbiota of Amynthas aspergillum across the seven habitats were dominated by *Pseudomonadota*, *Actinomycetota*, Chloroflexota, and *Bacillota* (Figure 7, Table 2). However, significant inter-group differences were observed in both their relative and absolute abundances.

In terms of relative abundance, *Actinomycetota* was overwhelmingly dominant in the HP and RGL groups, accounting for 28.4% and 26.7%, respectively, whereas it represented only 8.2% in the YZ group. *Pseudomonadota* reached its highest proportion in the HP group (22.1%) and its lowest in the PJ group (8.4%). *Bacillota* showed relatively high abundance in the RGL and HP groups (18.6% and 16.3%, respectively), but was relatively low in the YZ group (9.5%) (Figure 7).

Regarding absolute abundance (Table 2), *Actinomycetota* peaked in the RGL and HP groups, reaching 6.88 × 10^8^ copies/g and 6.68 × 10^8^ copies/g, respectively, compared to only 8.52 × 10^6^ copies/g in the YZ group. Chloroflexota exhibited the highest absolute abundance in the RGL group (3.89 × 10^8^ copies/g) and the lowest in the PJ and YZ groups. Similarly, the absolute abundance of *Bacillota* was highest in the RGL and HP groups (2.48 × 10^8^ copies/g and 1.65 × 10^8^ copies/g, respectively), while remaining below 4.0 × 10^6^ copies/g in both the FW and YZ groups.

### 3.5. Analysis of Relative/Absolute Abundance of Gut Microbiota at the Genus Level

**Figure 8:**
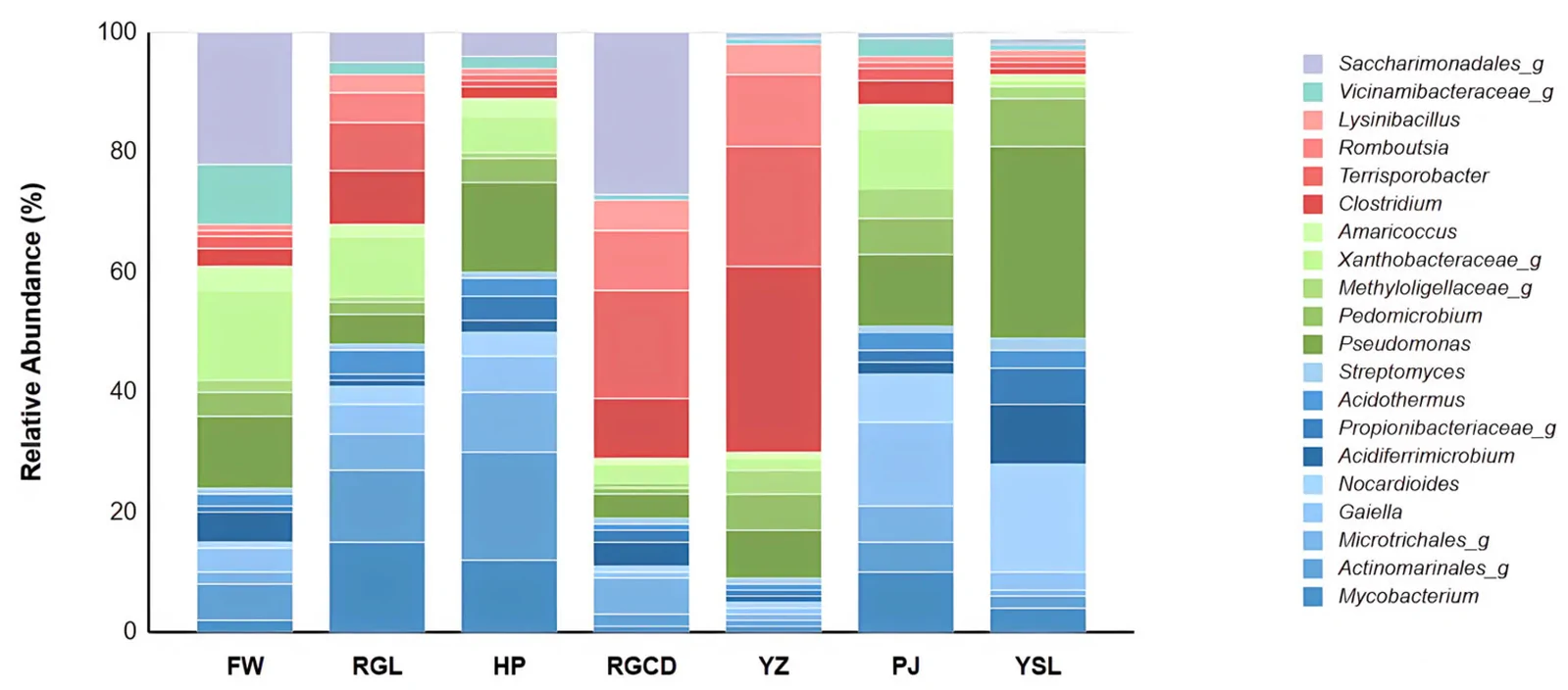
Relative abundance of major bacterial genera in the gut microbiota of *Amyntas aspergillum* across different habitats. The stacked bar chart displays the top 20 most abundant genera. FW, fertile farmland; HP, wasteland slope; PJ, barren farmland; RGCD, artificial grassland; RGL, plantation forest; YSL, primary forest; YZ, breeding farm.

At the genus level, the community composition exhibited stronger habitat specificity. In terms of relative abundance, *Mycobacterium* was most abundant in the RGL and RGCD groups, accounting for 8.4% and 7.2%, respectively (Figure 8). A genus within *Saccharimonadales* was the dominant taxon in the RGCD group with a relative abundance of 6.8%, which was significantly higher than in all other groups (all <1.0%). *Clostridium* showed the highest relative abundance in the RGL group (6.1%) and a relatively lower abundance in the YZ group (1.2%). *Pseudomonas* was relatively more abundant in the RGCD and HP groups, at 4.5% and 3.9%, respectively.

Regarding absolute abundance (copies/g), the absolute abundance of most dominant genera in the RGL and HP groups was significantly higher than in the other groups (Table 3). Specifically, the absolute abundance of *Mycobacterium* peaked in the RGL group (1.21 × 10^8^ copies/g), followed by the RGCD (3.30 × 10^7^ copies/g) and HP (2.45 × 10^7^ copies/g) groups, whereas it was significantly lower in the YZ group (1.77 × 10^6^ copies/g). *Saccharimonadales_genus* exhibited the highest absolute abundance in the RGCD group (1.30 × 10^8^ copies/g), far exceeding that in the other groups (HP: 2.90 × 10^7^ copies/g; RGL: 3.80 × 10^7^ copies/g), and remaining below 1.0 × 10^5^ copies/g in both the FW and PJ groups. The absolute abundance of *Clostridium* was highest in the RGL group (7.10 × 10^7^ copies/g), followed by the HP (1.35 × 10^7^ copies/g) and RGCD (5.20 × 10^6^ copies/g) groups, while it was only 1.78 × 10^5^ copies/g in the YZ group. Similarly, the absolute abundance of *Terrisporobacter* peaked in the RGL group (3.73 × 10^7^ copies/g), with all other groups showing values below 2.0 × 10^6^ copies/g.

### 3.6. Screening of LEfSe Differential Indicator Species (Biomarkers) of Gut Microbiota Across Seven Habitats

**Figure 9:**
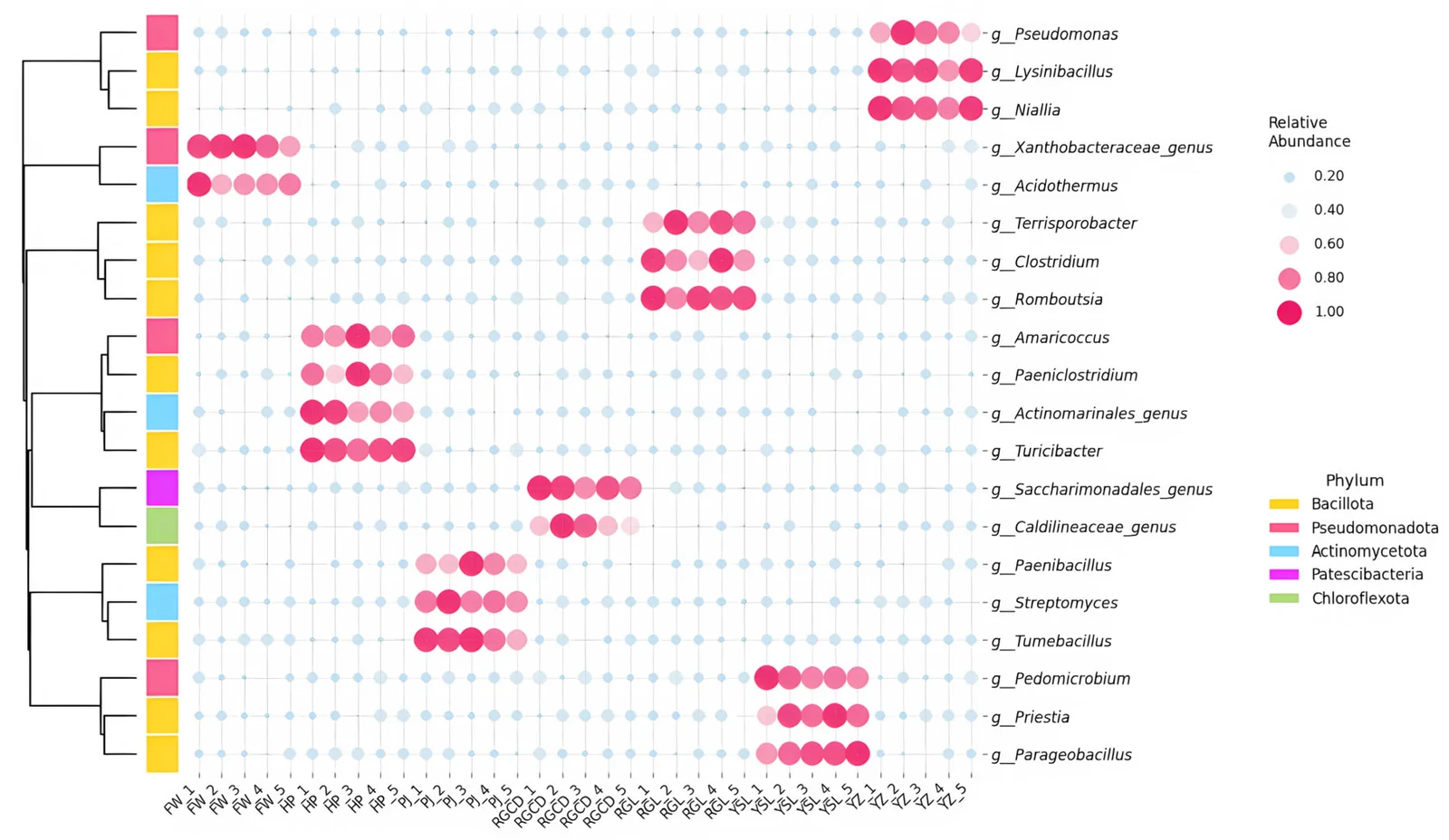
Discriminatory indicator taxa of gut microbiota in *Amyntas aspergillum* across different habitats identified by LEfSe analysis (LDA > 3.5, *p* < 0.05). FW, fertile farmland; HP, wasteland slope; PJ, barren farmland; RGCD, artificial grassland; RGL, plantation forest; YSL, primary forest; YZ, breeding farm.

Linear discriminant analysis effect size (LEfSe; LDA > 3.5,) delineated precise microbial biomarkers that serve as functional fingerprints of environmental filtering (Figure 9). Rather than a stochastic assemblage, the *A. aspergillum* gut microbiota is deterministically sculpted to meet the metabolic demands of its localized microhabitat.

In natural and semi-natural ecosystems characterized by complex or recalcitrant organic substrates, the host gut strategically enriched keystone degraders. For instance, the HP microenvironment specifically selected for *Streptomyces* and *Paenibacillus*, while the YSL favored taxa including *Amaricoccus* and *Turicibacter*. Agricultural environments shifted this evolutionary trajectory towards distinct metabolic specialists: FW was robustly characterized by *Acidothermus*, whereas PJ favored fermentative genera such as *Clostridium* and *Romboutsia*.

Intensive anthropogenic management drove the proliferation of highly specialized, stress-tolerant, or opportunistic indicator taxa. The RGCD group was profoundly characterized by the enrichment of *Pseudomonas* and *Lysinibacillus*. Conversely, the heavily managed YZ ecosystem was uniquely defined by *Pedomicrobium*, *Priestia*, and *Parageobacillus*. These distinct, non-overlapping indicator profiles—visually manifesting as high-abundance nodes exclusive to their respective environments—robustly confirm that habitat heterogeneity is the primary architect of core gut microbial composition and function.

### 3.7. Functional Prediction of Amynthas aspergillum Gut Microbiota

**Figure 10:**
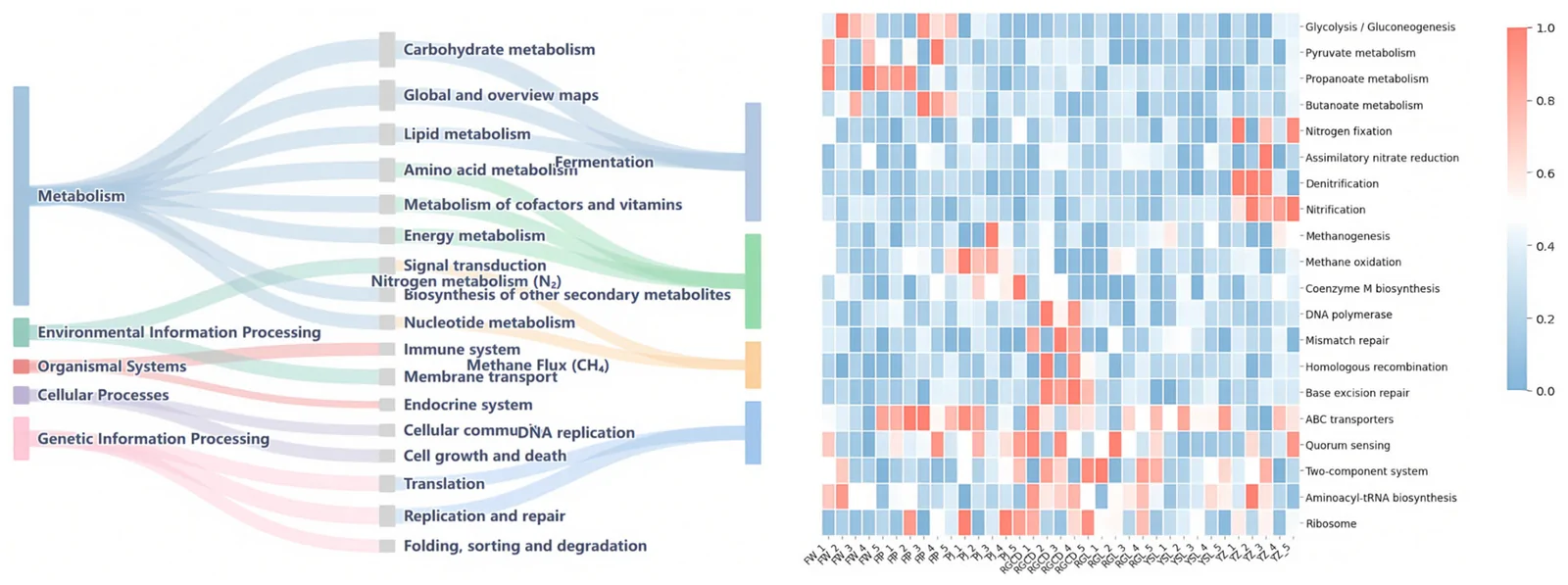
Functional profiling of *Amyntas aspergillum* gut microbiota based on KEGG database annotations. The left panel is a Sankey diagram illustrating the hierarchical flow of functional categories from Level 1 (e.g., Metabolism) to Level 3 (e.g., Carbohydrate metabolism). FW, fertile farmland; HP, wasteland slope; PJ, barren farmland; RGCD, artificial grassland; RGL, plantation forest; YSL, primary forest; YZ, breeding farm.

Based on PICRUSt2 predictions derived from 16S rRNA gene sequences and KEGG database annotations, the functional profiles of the gut microbiota of *A. aspergillum* under different habitat conditions exhibited a pattern of “core consistency with detailed differentiation.” (Figure 10). At the KEGG Level 1 and Level 2 pathway levels, the distribution patterns of gut microbiota across all habitat groups—including natural forests, plantations, wasteland slopes, farmlands, and breeding farms—were largely convergent. Metabolism was the most abundant functional category, followed by Genetic information processing, Environmental information processing, and Cellular processes. Key biological processes such as Carbohydrate metabolism, Amino acid metabolism, Energy metabolism, and ABC transporters maintained high abundances across all groups, constituting a stable functional framework for the gut micro-ecosystem. Further focusing on Level 3 pathways with significant differences, specific metabolic capabilities varied distinctively among groups, Natural and Semi-natural Habitats: Gut microbiota in primary forests, plantations, and wasteland slopes showed relatively higher predicted abundances in pathways related to Aromatic compound degradation, Lignin degradation, Bacterial chemotaxis, and the biosynthesis of certain secondary metabolites. Artificial Breeding Habitats: These presented a different functional emphasis, with significantly higher predicted abundances in Fermentation, Propanoate metabolism, and pathways related to specific carbohydrate degradation compared to other groups. Conversely, they exhibited lower abundances in pathways such as Lignin degradation and Bacterial chemotaxis. Farmland Habitats: Fertile and barren farmlands displayed intermediate abundance levels in nitrogen metabolism-related pathways, such as Nitrogen fixation, and specific sugar metabolism pathways, falling between natural and breeding habitats. Additionally, numerical differences were observed between these two groups in certain subtle metabolic branches. In summary, while the core Level 1 and Level 2 metabolic functions of the gut microbiota remained relatively stable across different habitats, significant abundance differences were observed at the Level 3 pathway level. These differences were particularly evident in pathways involving specific substrate degradation (e.g., Lignin degradationvs. easily fermentable carbon sources) and environmental responses (e.g., Bacterial chemotaxis).

## 4. Discussion

### 4.1. Absolute and Relative Differences in Structure and Diversity of Amynthas aspergillum Gut Microbiota Across Seven Habitats

This study, integrating relative abundance with absolute quantification based on 16S rRNA gene copy numbers, comprehensively revealed the highly significant impact of habitat type on the α- and β-diversity of the gut microbiota of *A. aspergillum* (Figure 5 and Figure 6). Absolute quantification results indicated that the FW group not only possessed the highest α-diversity indices (Chao1 index: 2500~4000; Shannon index: ~7.0) but also maintained high absolute abundances (measured in sequencing copies) for multiple dominant phyla. For instance, the copy number of *Actinomycetota* in the FW group was 1.49 × 10^8^ copies/g, significantly higher than that in the YZ group (Table 2). This confirms that the fertile farmland habitat provides abundant colonization resources and ecological niches for microorganisms.

In contrast, the YZ group exhibited a low overall Shannon diversity index alongside noticeable fluctuations in community evenness. Combined with absolute quantitative data, although the copy number of *Bacillota* in the YZ group (3.59 × 10^6^ copies/g) was significantly lower than in the HP or RGL groups, the copy number of its characteristic genus, *Clostridium*, reached as high as 7.10 × 10^7^ copies/g in the RGL group (Table 3). Furthermore, in the YZ group, biomarkers identified by LEfSe analysis (Figure 9), such as *Lysinibacillus* and *Pseudomonas,* were also significantly enriched. This quantitatively corroborates the conclusion that the community structure in this habitat is simplified, with specific species occupying an absolute dominance. This finding aligns with the view of Ortiz-Gamino et al. that land use type acts as a key abiotic filter driving earthworm community structure [34]. By utilizing copy number-based absolute quantification, this study is the first to quantify the filtering intensity of different anthropogenic management activities—such as single-substrate farming—on earthworm gut microbiota from the perspective of absolute microbial load.

The microbial community structure of the RGCD group was the most distinct in terms of β-diversity (Figure 6). Moreover, the copy numbers of several dominant phyla (e.g., *Actinomycetota*: 2.39 × 10^8^ copies/g; Chloroflexota: 1.84 × 10^8^ copies/g) were at moderate to low levels (Table 2). This is consistent with the phenomenon of biotic homogenization caused by agricultural intensification observed by Tsiafouli et al. [35]. Absolute quantitative analysis further revealed that this structural difference stems from the trade-off in absolute numbers of specific taxa rather than a simple increase or decrease in total load [36]. For example, the copy number of *Saccharimonadales_genus* in the RGCD group reached 1.30 × 10^8^ copies/g (Table 3), becoming its absolute dominant taxon, while the numbers of other taxa were relatively restricted. The fundamental reason may lie in the strict “environmental filtering” imposed by the highly homogeneous microenvironment (frequent mowing, single grass species) on ingested microorganisms [37].

Meanwhile, although the substrate of the YZ group had a high organic matter content (20.72 g/kg, Table 1), the α-diversity of its gut microbiota did not increase correspondingly, and its Gini-Simpson index values remained confined at a lower numerical scale as reflected in the shifting niche patterns. Absolute quantification results explained the phenomenon that although the total quantity of certain phyla in the YZ group (e.g., *Pseudomonadota*, with an absolute abundance of 4.86 × 10^6^ copies/g) was considerable, specific genera closely related to environmental characteristics—such as high organic load and potential resistance pressure—like *Lysinibacillus* and *Pseudomonas*, were numerically dominant. This led to a highly specialized and dominant ecological niche pattern where the gut microbiota was functionally highly simplified and dominated by a few taxa. This phenomenon is similar to the trend of earthworm community simplification observed by Pelosi et al. in long-term disturbed forest soils [38], suggesting that high-density farming models with single feed sources (fermented cow dung) may cause “eutrophication stress” by providing excessive but monotonous types of easily degradable carbon sources. This inhibits the growth and competition of other functional microbial communities in terms of absolute numbers, leading to a decline in the functional redundancy of the microbial community, which is also consistent with Louca et al.’s assertion that the loss of microbial functional redundancy weakens ecosystem resilience [39]. The seemingly paradoxical low C/N ratio (9.38) observed at the YZ site, despite straw inclusion, is heavily attributed to the intense pre-fermentation process where labile carbon was rapidly respired by hyperactive microbes, leaving a nitrogen-dense consolidated matrix.

### 4.2. Habitat-Specific Succession and Functional Response of Dominant Gut Microbiota in Amynthas aspergillum

Through cross-validation of relative and absolute abundances, this study more accurately characterized the habitat-specific succession of dominant microbial communities (Figure 7 and Figure 8; Table 2 and Table 3). At the phylum level, *Actinomycetota*, *Pseudomonadota*, Chloroflexota, and *Bacillota* were dominant across all groups; however, their absolute abundances (copy numbers) exhibited significant gradient variations. For instance, in the HP and RGL groups, the copy numbers of *Actinomycetota* reached 6.68 × 10^8^ copies/g and 6.88 × 10^8^ copies/g, respectively, while Chloroflexota reached as high as 2.11 × 10^8^ copies/g and 3.89 × 10^8^ copies/g (Table 2), significantly higher than in other habitats such as the FW group.

This absolute quantitative evidence strongly supports the inference that in habitats with relatively poor soil (HP group organic matter: 7.02 g/kg) or rich in recalcitrant lignin (RGL group), the gut micro-ecosystem of *A. aspergillum* tends to recruit and maintain high quantities of “specialist” microorganisms with powerful catabolic capabilities [40,41,42,43,44]. Goswomi et al. emphasized the key role of *Actinomycetota* in degrading recalcitrant organic matter (e.g., chitin, lignin) [45], while Zhang et al. pointed out the important role of Chloroflexota in the transformation and fixation of recalcitrant carbon components in soil. This microbial structure represents a key strategy for the host to actively adapt to harsh nutritional environments and maximize energy acquisition efficiency [46]. Conversely, in fertile farmland (FW), while “opportunist” bacteria such as *Pseudomonadota* showed high relative abundance, their copy numbers (1.61 × 10^7^ copies/g) did not form the overwhelming dominance seen in HP or RGL groups (Table 2). This reflects that in this habitat (organic matter: 29.12 g/kg), soil nutrients are sufficient and resource heterogeneity is high; consequently, the microbial community flourishes overall, and interspecific competition is relatively moderate. This aligns with the ecological characteristics described by Babbin et al., where many *Pseudomonadota* taxa are r-strategists that proliferate rapidly when resources are abundant [47].

The advantage of absolute quantitative analysis is particularly prominent at the genus level, as it effectively avoids the misleading “dilution effect” that may arise from differences in total bacterial load among samples in relative abundance analysis [14,48,49,50,51]. The ecological indicative significance of the habitat-specific biomarkers identified by LEfSe analysis (Figure 9) is firmly supported by absolute quantitative data. For example, the enrichment of biomarkers Lysinibacillus and Pseudomonas in the YZ group is not an artifact of relative proportions. Although Table 3 does not directly list the copy numbers for these genera in the YZ group, their high LDA values in LEfSe analysis, combined with references from other groups (e.g., *Pseudomonas* at 2.75 × 10^7^ copies/g in the HP group), allow us to infer their significant absolute numerical dominance in the YZ group. This not only reflects the high protein and high ammonia nitrogen characteristics of the breeding substrate (cow dung) but also confirms, in terms of absolute numbers, the competitive advantage of these genera in coping with specific selection pressures in intensive farming environments (such as residual veterinary antibiotics or heavy metals in feed). Zhao et al. warned that the agricultural reuse of livestock manure is an important pathway for the spread of antibiotic resistance genes in the environment [52], and Litvak et al. observed a significant increase in the absolute abundance of specific potential pathogens in the gut under disease states in fecal microbial models [53], which echoes the ecological selection mechanism found in this study. This integrated analysis method, combining absolute quantification, enables us to go beyond the appearance presented by relative abundance and more precisely identify the key microbial taxa that truly dominate gut ecological processes at the cellular numerical level in specific habitats.

### 4.3. Adaptation of Amynthas aspergillum Gut Microbiota Function to Habitat Stress

Based on PICRUSt2 functional predictions and the associated absolute quantitative context, we have gained a deeper understanding of the differences in the metabolic potential of gut microbiota across different habitats (Figure 10). In the HP and RGL groups, pathways related to “Environmental Information Processing” (e.g., bacterial chemotaxis, two-component systems) and “Biosynthesis of antibiotics” showed higher predicted abundances. It is crucial to emphasize that absolute quantitative data indicates that the microbial cells executing these functional potentials (such as enriched *Actinomycetota* and Chloroflexota-related taxa) maintain extremely high copy numbers in these habitats (Table 2). This implies that under the stress of barrenness or complex organic matter, the gut microbiota not only tends to enhance environmental sensing and competitive defense capabilities at the level of the gene functional profile but also possesses an abundance of microbes capable of executing these “survival defense” strategies. This represents a comprehensive adaptation from gene potential to cellular entity. Carey et al. elucidated that bacterial chemotaxis systems are a core mechanism for bacteria to sense environmental gradients and actively locate scarce nutrient sources to cope with nutritional stress [54], while Sharma et al. demonstrated that antibiotic production is an effective strategy for niche defense in competitive environments. The high absolute bacterial load and the enrichment of corresponding functional pathways in the HP and RGL groups in this study corroborate this adaptive restructuring.

In nutrient-rich fertile farmland (FW) and breeding farms (YZ), the microbial functional profile is noticeably tilted towards “basic substance metabolism” (e.g., carbohydrate metabolism such as glycolysis and the pentose phosphate pathway). Absolute quantitative analysis reveals a key phenomenon: taking the YZ group as an example, although it carries a considerable total microbial cell load (inferred from dominant phylum abundance), its functional profile exhibits a distinct “simplification” trend—meaning functional diversity does not increase in tandem with microbial numbers. In the YZ group, the high number of microbial cells may be highly concentrated on a few highly efficient energy-producing metabolic pathways (e.g., basic sugar metabolism) to rapidly utilize single, easily degradable carbon sources, leading to a decline in the elasticity of the overall metabolic network. The concept of microbial community functional redundancy proposed by Zelezniak et al. highlights its key role as an “insurance mechanism” for ecosystems to cope with disturbances, whereas the simplification of the functional profile implies a loss of this redundancy, rendering the system more vulnerable [55]. This explains the micro-ecological mechanisms behind problems often seen in *A. aspergillum* in intensive farming, such as poor disease resistance and growth fluctuations, from the perspective of the mismatch between “microbial quantity” and “functional diversity” [56,57,58,59]: a gut ecosystem dominated by extremely high numbers of functionally convergent microbiota is low in stability and resilience [60,61,62]. By simulating complex feed formulations from natural habitats, we can cultivate a gut microbial ecosystem that is balanced in absolute numbers and diverse and complementary in function. This concept aligns with the strategy advocated by Clagnan et al. of designing synthetic microbial communities based on the principle of functional complementarity to enhance system stability [63]. By integrating absolute quantitative analysis, this study advances gut microbiota research from the traditional level of relative composition to a new level linking absolute quantity with ecological function, providing a more solid and refined quantitative basis for scientifically assessing earthworm health and precisely regulating their farming microenvironment.

## 5. Conclusions

There is a minimal overlap of core microbiota shared among different habitats, whereas significant differences exist in community structure and diversity. This confirms that soil physicochemical properties (pH, organic matter, C/N ratio) and anthropogenic disturbance constitute key environmental filters that drive the deterministic assembly of gut microbiota. Wasteland slopes and plantations enrich *Actinomycetota* (e.g., *Streptomyces*) that are adept at degrading recalcitrant organic matter. In contrast, breeding farms specifically enrich *Bacillota* (e.g., *Clostridium*) capable of anaerobic fermentation, as well as stress-resistant *Pseudomonadota* (e.g., *Pseudomonas*). This indicates that specific habitats enrich microbial communities that are functionally matched to them. Microbiota in natural habitats exhibit enhanced complex functions such as environmental sensing and competitive defense, whereas simplified habitats like breeding farms lead to a “simplification” of microbiota functions, concentrating them on basic sugar metabolism. This suggests that anthropogenic intervention may result in a decline in the functional redundancy of the microbiota.

## Figures and Tables

**Table 1:** Soil nutrient and physicochemical properties under different habitats.

Item	Group							SEM	*p*-Value
	FW	HP	PJ	RGCD	RGL	YSL	YZ		
pH	6.78 ^d^	8.02 ^e^	6.10 ^bc^	6.56 ^cd^	6.02 ^b^	5.34 ^a^	6.84 ^d^	0.16	<0.001
Organic Matter (g/kg)	29.12 ^f^	7.02 ^a^	12.54 ^b^	22.72 ^de^	18.20 ^c^	24.16 ^e^	20.72 ^d^	1.04	<0.001
Total Nitrogen (g/kg)	2.10 ^e^	0.42 ^a^	0.72 ^b^	1.44 ^d^	1.00 ^c^	1.22 ^c^	1.24 ^c^	0.08	<0.001
Total Phosphorus (g/kg)	0.98 ^f^	0.32 ^a^	0.38 ^b^	0.78 ^e^	0.50 ^c^	0.60 ^d^	1.00 ^g^	0.05	<0.001
Total Potassium (g/kg)	18.30 ^e^	7.06 ^a^	10.00 ^b^	17.20 ^d^	12.70 ^c^	14.80 ^c^	14.90 ^c^	0.85	<0.001
Carbon-to-Nitrogen Ratio (C/N)	10.90 ^c^	17.70 ^f^	14.50 ^d^	12.00 ^b^	15.40 ^e^	16.90 ^f^	9.38 ^a^	0.58	<0.001
Electrical Conductivity (μS/cm)	197.00 ^a^	407.00 ^c^	92.60 ^b^	119.00 ^d^	90.60 ^b^	80.70 ^b^	320.00 ^e^	13.50	<0.001

FW, fertile farmland; HP, wasteland slope; PJ, barren farmland; RGCD, artificial grassland; RGL, plantation forest; YSL, primary forest; YZ, breeding farm. Different superscript lowercase letters within the same row indicate significant differences among groups according to Duncan’s multiple range test (*p* < 0.05).

**Table 2:** Comparison of absolute abundances (in copies) of dominant bacterial phyla in the gut of *Amynthas aspergillum* under different habitat conditions.

Phylum	Groups							SEM	*p*-Value
	FW	HP	PJ	RGCD	RGL	YSL	YZ		
*Actinomycetota*	1.49 × 10^8 b^	6.68 × 10^8 a^	5.82 × 10^6 c^	2.39 × 10^8 b^	6.88 × 10^8 a^	3.00 × 10^7 c^	8.52 × 10^6 c^	4.10 × 10^7^	<0.001
Chloroflexota	2.14 × 10^7 c^	2.11 × 10^8 b^	5.25 × 10^6 c^	1.84 × 10^8 b^	3.89 × 10^8 a^	1.57 × 10^7 c^	4.73 × 10^6 c^	2.40 × 10^7^	<0.001
*Pseudomonadota*	1.61 × 10^7 c^	3.82 × 10^8 a^	1.28 × 10^6 d^	2.01 × 10^7 c^	2.58 × 10^8 b^	2.78 × 10^7 c^	4.86 × 10^6 cd^	2.30 × 10^7^	<0.001
*Bacillota*	3.72 × 10^6 c^	1.65 × 10^8 a^	3.14 × 10^6 c^	2.90 × 10^7 bc^	2.48 × 10^8 a^	6.62 × 10^6 c^	3.59 × 10^6 c^	1.30 × 10^7^	<0.001
Patescibacteria	7.50 × 10^5 c^	6.32 × 10^7 b^	2.55 × 10^5 c^	1.55 × 10^8 a^	6.92 × 10^7 b^	2.65 × 10^6 c^	1.51 × 10^6 c^	1.10 × 10^7^	<0.001
Planctomycetota	2.31 × 10^6 c^	1.88 × 10^8 a^	5.61 × 10^5 c^	5.07 × 10^6 c^	6.75 × 10^7 b^	4.96 × 10^6 c^	6.73 × 10^5 c^	1.20 × 10^7^	<0.001
Acidobacteriota	3.96 × 10^6 b^	6.98 × 10^7 a^	3.50 × 10^5 c^	1.26 × 10^5 c^	8.05 × 10^6 b^	1.78 × 10^6 c^	7.69 × 10^5 c^	4.20 × 10^6^	<0.001
Myxococcota	6.97 × 10^5 c^	2.05 × 10^7 a^	1.81 × 10^5 c^	5.04 × 10^5 c^	5.14 × 10^7 a^	3.29 × 10^6 b^	4.00 × 10^5 c^	3.10 × 10^6^	0.002
Chlamydiota	4.80 × 10^5 c^	1.42 × 10^7 a^	1.64 × 10^5 c^	4.98 × 10^6 b^	2.80 × 10^7 a^	1.20 × 10^6 bc^	6.56 × 10^5 c^	1.90 × 10^6^	0.008
Verrucomicrobiota	6.90 × 10^5 c^	3.00 × 10^7 a^	2.52 × 10^5 c^	2.89 × 10^5 c^	9.78 × 10^6 b^	4.15 × 10^5 c^	5.83 × 10^5 c^	1.10 × 10^6^	<0.001

FW, fertile farmland; HP, wasteland slope; PJ, barren farmland; RGCD, artificial grassland; RGL, plantation forest; YSL, primary forest; YZ, breeding farm. Different lowercase letters within the same row indicate significant differences among groups (*p* < 0.05).

**Table 3:** Comparison of absolute abundances (in copies) of dominant bacterial genus in the gut of *Amynthas aspergillum* under different habitat conditions.

Genus	Groups							SEM	*p*-Value
	FW	HP	PJ	RGCD	RGL	YSL	YZ		
*Mycobacterium*	9.37 × 10^5 c^	2.45 × 10^7 a^	9.77 × 10^5 c^	3.30 × 10^7 a^	1.21 × 10^8 b^	3.83 × 10^6 c^	1.77 × 10^6 c^	1.69 × 10^7^	0.0002
*Saccharimonadales_genus*	9.73 × 10^4 c^	2.90 × 10^7 a^	4.35 × 10^4 c^	1.30 × 10^8 b^	3.80 × 10^7 a^	5.22 × 10^5 c^	5.27 × 10^4 c^	1.25 × 10^7^	<0.0001
*Clostridium*	2.40 × 10^5 c^	1.35 × 10^7 a^	2.28 × 10^5 c^	5.20 × 10^6 b^	7.10 × 10^7 a^	1.47 × 10^5 c^	1.78 × 10^5 c^	9.73 × 10^6^	0.0001
*Pseudomonas*	2.63 × 10^5 c^	2.75 × 10^7 a^	3.94 × 10^5 c^	1.24 × 10^6 bc^	1.97 × 10^7 a^	9.39 × 10^5 b^	7.29 × 10^5 bc^	4.70 × 10^6^	0.0004
*Terrisporobacter*	1.65 × 10^3 c^	1.49 × 10^6 ab^	1.49 × 10^4 c^	1.93 × 10^6 ab^	3.73 × 10^7 a^	9.54 × 10^2 c^	1.75 × 10^4 c^	4.90 × 10^6^	<0.0001
*Actinomarinales_genus*	6.26 × 10^3 c^	3.10 × 10^8 a^	0.00 ^d^	8.24 × 10^5 c^	6.24 × 10^6 b^	6.74 × 10^3 c^	5.98 × 10^2 c^	2.39 × 10^7^	<0.0001
*Microtrichales_genus*	1.70 × 10^5 c^	1.24 × 10^7 ab^	8.08 × 10^3 c^	3.36 × 10^7 a^	3.87 × 10^7 a^	4.07 × 10^5 bc^	5.71 × 10^4 c^	5.98 × 10^6^	<0.0001
*Gaiella*	1.13 × 10^5 c^	2.79 × 10^7 a^	7.88 × 10^4 c^	5.54 × 10^5 bc^	5.45 × 10^7 a^	5.65 × 10^5 bc^	3.75 × 10^4 c^	6.99 × 10^6^	<0.0001
*Nocardioides*	5.56 × 10^4 c^	1.89 × 10^7 a^	2.69 × 10^5 c^	2.86 × 10^6 b^	4.66 × 10^7 a^	7.62 × 10^5 bc^	4.45 × 10^5 c^	5.55 × 10^6^	<0.0001
*Romboutsia*	1.86 × 10^4 c^	3.33 × 10^7 a^	8.02 × 10^4 c^	7.10 × 10^6 b^	2.55 × 10^7 a^	3.17 × 10^4 c^	7.85 × 10^4 c^	4.37 × 10^6^	<0.0001
*Pedomicrobium*	9.31 × 10^5 c^	2.85 × 10^7 a^	1.22 × 10^4 d^	9.74 × 10^4 cd^	3.03 × 10^7 a^	6.05 × 10^6 b^	1.71 × 10^5 c^	5.83 × 10^6^	0.0006
*Methyloligellaceae_genus*	3.78 × 10^5 c^	3.69 × 10^7 a^	7.71 × 10^4 c^	2.58 × 10^5 c^	2.61 × 10^7 a^	1.74 × 10^6 b^	4.03 × 10^4 c^	4.49 × 10^6^	<0.0001
*Acidiferrimicrobium*	1.60 × 10^6 c^	1.33 × 10^7 a^	2.77 × 10^5 d^	9.12 × 10^6 b^	2.75 × 10^7 a^	1.57 × 10^6 c^	4.69 × 10^5 cd^	3.62 × 10^6^	0.0001
*Vicinamibacteraceae_genus*	1.66 × 10^3 c^	3.55 × 10^7 a^	0.00 ^d^	0.00 ^d^	2.50 × 10^6 b^	4.77 × 10^4 c^	0.00 ^d^	2.75 × 10^6^	<0.0001
*Propionibacteriaceae_genus*	4.80 × 10^2 c^	1.11 × 10^7 a^	6.46 × 10^1 c^	3.06 × 10^6 b^	2.32 × 10^7 a^	1.72 × 10^4 c^	0.00 ^c^	2.75 × 10^6^	<0.0001

FW, fertile farmland; HP, wasteland slope; PJ, barren farmland; RGCD, artificial grassland; RGL, plantation forest; YSL, primary forest; YZ, breeding farm. Different lowercase letters within the same row indicate significant differences among groups (*p* < 0.05).

## Data Availability

The raw 16S rRNA gene microbial amplicon sequencing datasets generated during the current study have been deposited in the NCBI Sequence Read Archive (SRA) repository under the project tracking identifier 25B0227D-1. The datasets are available from the corresponding author upon reasonable request.
